# The COVID-19 Vaccines: Recent Development, Challenges and Prospects

**DOI:** 10.3390/vaccines9040349

**Published:** 2021-04-05

**Authors:** Yuxin Yan, Yoongxin Pang, Zhuoyi Lyu, Ruiqi Wang, Xinyun Wu, Chong You, Haitao Zhao, Sivakumar Manickam, Edward Lester, Tao Wu, Cheng Heng Pang

**Affiliations:** 1 Department of Chemical and Environmental Engineering, University of Nottingham Ningbo China, Ningbo 315100, China; yuxin.yan@nottingham.edu.cn (Y.Y.); slyzl6@nottingham.edu.cn (Z.L.); tao.wu@nottingham.edu.cn (T.W.); 2 New Materials Institute, University of Nottingham Ningbo China, Ningbo 315042, China; yoongxin.pang@nottingham.edu.cn (Y.P.); shyrw2@nottingham.edu.cn (R.W.); xinyun.wu@nottingham.edu.cn (X.W.); 3 Beijing International Center for Mathematical Research, Peking University, Beijing 100871, China; chongy@pku.edu.cn; 4 MITMECHE, Massachusetts Institute of Technology, Cambridge, MA 02139, USA; haitaoz@mit.edu; 5 Petroleum and Chemical Engineering, Faculty of Engineering, Universiti Teknologi Brunei, Bandar Seri Begawan BE1410, Brunei; manickam.sivakumar@utb.edu.bn; 6 Department of Chemical and Environmental Engineering, University of Nottingham, Nottingham NG7 2RD, UK; edward.lester@nottingham.ac.uk; 7 Key Laboratory for Carbonaceous Wastes Processing and Process Intensification Research of Zhejiang Province, University of Nottingham Ningbo China, Ningbo 315100, China; 8 Municipal Key Laboratory of Clean Energy Conversion Technologies, University of Nottingham Ningbo China, Ningbo 315100, China

**Keywords:** vaccines, COVID-19, global pandemic, coronavirus, SARS-CoV-2

## Abstract

The highly infectious coronavirus disease 2019 (COVID-19) associated with the pathogenic severe acute respiratory syndrome coronavirus 2 (SARS-CoV-2) has spread to become a global pandemic. At present, the world is relying mainly on containment and hygiene-related measures, as well as repurposed drugs to control the outbreak. The development of COVID-19 vaccines is crucial for the world to return to pre-pandemic normalcy, and a collective global effort has been invested into protection against SARS-CoV-2. As of March 2021, thirteen vaccines have been approved for application whilst over 90 vaccine candidates are under clinical trials. This review focuses on the development of COVID-19 vaccines and highlights the efficacy and vaccination reactions of the authorised vaccines. The mechanisms, storage, and dosage specification of vaccine candidates at the advanced stage of development are also critically reviewed together with considerations for potential challenges. Whilst the development of a vaccine is, in general, in its infancy, current progress is promising. However, the world population will have to continue to adapt to the “new normal” and practice social distancing and hygienic measures, at least until effective vaccines are available to the general public.

## 1. Introduction

The ongoing pandemic of coronavirus disease (COVID-19) has impacted the world socially and economically on many different levels. COVID-19 is caused by a new strain of coronavirus known as severe acute respiratory syndrome coronavirus-2 (SARS-CoV-2), with a much higher transmission rate of around 2.2 per patient within China [1]. Phylogenetic analysis of the full sequence of SARS-CoV-2 suggests that the virus belongs to the subgenus Sarbecovirus in genus lineage B betacoronavirus genre [2] as the gene sequence of SARS-CoV-2 is 89% identical to bat SARS-like coronavirus ZXC21 (bat-SL-CoVZXC21, accession no. MG772934.1) and ZC45 (MG772933.1); and it is 82% identical to SARS-CoV Tor2 (JX163927) [3]. Though its nucleotide has shown 96.2% similarity with the one of bat coronavirus at the whole genome level, the primary reservoir of SARS-CoV-2 has not been determined [4]. Researchers investigated the crystal structure of the C-terminal domain of SARS-CoV-2 (SARS-CoV-2-CTD) spike (S) protein in complex with human angiotensin-converting enzyme 2 (hACE2) and reported that SARS-CoV-2-CTD exhibited a stronger interaction at the binding interface as compared to SARS-receptor-binding domain (SARS-RBD) [5]. The binding profiles and affinity constants (K_D_), i.e., SARS-CoV-2-S1 and SARS-CoV-2-CTD were recorded as 94.6 ± 6.5 nM and 133.3 ± 5.6 nM, respectively. Meanwhile, SARS-RBD engaging the same receptor was recorded as 408.7 ± 11.1 nM [5], which revealed that the atomic interactions between hACE2 and SARS-CoV-2-CTD are approximately four times higher than that with SARS-RBD. SARS-CoV-2 is both pathogenic and zoonotic, thereby allowing the transmission between animals and humans, leading to epidemic regionally and globally [3]. Research on the transmission dynamics of the SARS-CoV-2 revealed the transmissibility of SARS-CoV-2 is within the range of 0.8–5.7 [6,7,8]. More than a year since the detection of SARS-CoV-2, there have been over 121 million confirmed cases recorded and the disease has claimed over 2.7 million lives around the world as recorded on 20 March 2021. As the world population is adapting to what is being called the “new normal” of social distancing, mask-wearing, and temperature screening, an effective medical approach seems to be the only route for life to return to what it was pre-pandemic. Many countries have employed the former with the help of advanced science and technology where scientists and healthcare experts have come together to accelerate potential prophylactic and curative treatment to curb the spread and detrimental effect of the virus in the form of new, effective treatments and vaccines.

## 2. Disease Manifestations and Principles

### 2.1. Onset Condition

The SARS-CoV-2 is a betacoronavirus, and current research infers that the natural host may be bats [9]. This virus is known to strongly infect human respiratory and intestinal epithelial cells, as well as the neurological and central nervous system through the molecular mechanism of interaction between spike (S) protein and ACE2 receptors in the human body [10,11]. Jing et al. proposed an estimated incubation period model, which shows the median incubation time of COVID-19 to be 7.76 days [95% confidence interval (CI): 7.02 to 8.53], while 95 percentile to be 14.28 days (95% CI: 13.64 to 14.90) [12]. Infected patients may or may not be symptomatic but are infectious nonetheless. Most of the signs and symptoms of COVID-19 are similar to other respiratory or viral illnesses which include fever, chills, cough, shortness of breath, fatigue, muscle aches, headache, sore throat, loss of smell and taste, and congestion or runny nose. These symptoms may vary in individuals and as the disease progresses. Most COVID-19 patients develop mild (40%) or moderate (40%) symptoms, approximately 15% develop a severe disease that requires oxygen support, and 5% develop the critical disease with complications including respiratory failure, acute respiratory distress syndrome (ARDS), sepsis and septic shock, thromboembolism, and/or multi-organ failure [13,14]. Patients with other comorbidities (underlying non-communicable diseases) are at a higher risk for the disease to progress to a more critical stage.

Epidemiological studies have shown that the main transmission route of SARS-CoV-2 is through close contact and human-to-human transmission which occurs through droplets, aerosols, and contact [10]. Hence, many countries (but not all) have practiced, encouraged, and even enforced lockdown, social distancing, and mask-wearing.

### 2.2. Detection of Coronavirus in Plasma

Nucleic acid testing is required to identify if a patient is infected by SARS-CoV-2. The positive result from the new highly specific coronavirus nucleic acid test confirms the diagnosis of COVID-19 [10,15]. Important pathogenic evidence for the diagnosis of COVID-19 infection, such as real-time fluorescent reverse transcriptase-polymerase chain reaction (RT-PCR) and gene sequencing technologies, have been included in the COVID-19 diagnosis and treatment specifications of the National Health Commission [16] to alleviate the impact of this pandemic on humans. Currently, nucleic acid detection based on the principle of RT-PCR is the main method for pathogen detection and disease diagnosis.

Antibody-based and genome-based detection methods are used to identify infections caused by SARS-CoV-2. The genome detection method provides a preliminary diagnosis, that is, identification by nasal swabs and throat swabs sampling. However, the sensitivity of such a detection method is relatively low. Antibody-based detection is a more sensitive approach performed by extracting and amplifying the viral RNA collected from the plasma of patients with abnormal body temperature, followed by qualitative and quantitative real-time determination if virus RNA is present in the patient [17]. In addition, the use of plasma viremia for diagnosis does not require nasopharyngeal aspiration, which avoids certain risks during specimen collection [18].

### 2.3. Treatment

Currently, there are no specific treatments for COVID-19 disease but clinical guidance for the management of suspected and confirmed COVID-19 patients are available for healthcare providers. However, these guidelines are not meant to replace clinical judgment or specialist consultation and are updated from time to time as new treatment options are available and approved based on research and clinical trial outcomes.

The pharmacological therapies generally administered in the management of COVID-19 patients are discussed.

#### 2.3.1. Mild to Moderate COVID-19 Symptomatic Patients

WHO recommends that patients with suspected or confirmed mild to moderate COVID-19 symptoms be isolated to prevent virus transmission and they are given symptomatic treatment such as antipyretics for fever and pain, adequate nutrition, and appropriate rehydration [19]. The use of anti-bacterial therapy or prophylaxis is discouraged in these patients as it will contribute to higher bacterial resistance rates unless there is a clinical suspicion of a bacterial infection.

The National Institutes of Health (NIH) of Unites States of America mentions the use of SARS-CoV-2-neutralising-antibodies (bamlanivimab or casirivimab plus imdevimab) in outpatients who are at high risk for disease progression [20]. Anti-SARS-CoV-2 antibody-based therapies may have an effect before the host has mounted an effective immune response. This was based on preliminary data results of the observed reduction in COVID-19-related hospitalisation or emergency room visits within 28 days after treatment when compared to placebo. The investigational monoclonal antibody therapy was issued an Emergency Use Authorization (EUA) by the United States Food and Drug Administration (USFDA) [21,22].

Patients being treated at home who developed symptoms with complications (e.g., difficulty in breathing, chest pain, etc.) should seek emergency help while patients in hospital should have close monitoring to recognise signs and symptoms of disease progression [23].

#### 2.3.2. Severe COVID-19 Patients

WHO recommends immediate administration of supplemental oxygen therapy and emergency airway management to any patient with emergency signs (obstructed or absent breathing, severe respiratory distress, central cyanosis, shock, coma, and/or convulsions) and to any patient without emergency signs but with oxygen saturation (SpO_2_) < 90%, to regulate the SpO_2_ to a normal level of ≥94% [23]. It is also recommended to ensure the SpO_2_ of pregnant patients is ≥92–95% whilst non-pregnant adults and child patients are >90%.

The only anti-viral drug approved or authorised for temporary use in over 50 countries for the treatment of COVID-19 in severe hospitalised patients is remdesivir, the RNA-dependent RNA-polymerase (RdRp) inhibitor of the Ebola virus and is expected to have broad-spectrum antiviral effects [24]. It is an adenosine nucleotide analogue that can interrupt virus replication. Remdesivir was granted approval by the USFDA on 22 October 2020 and conditional marketing authorisation by the European Commission for the treatment in COVID-19 patients for adults and paediatric patients (12 years of age and older and weighing at least 40kg) based on the study of the National Institute of Allergy and Infectious Diseases Adaptive COVID-19 Treatment Trial (NIAID-ACTT-1). Study showed that remdesivir improved recovery time (10 days with 95% confidence interval (95% CI: 9 to 11) compared with 15 days (95% CI: 13 to 18) in the placebo arm (rate ratio for recovery, 1.29; 95% CI: 1.12 to 1.49; log-rank *p*-value < 0.0001) [25]. This suggests the administration of remdesivir could reduce hospitalisation time especially in severe disease patients requiring supplemental oxygen.

Another anti-viral drug widely used is favipiravir, a nucleoside analog and is metabolised in cells to a ribosyl triphosphate form (favipiravir RTP) that selectively inhibits RNA polymerase involved in influenza viral replication. It is used as an antiviral treatment for influenza A and B [26]. There were two open-label clinical studies conducted in China, one comparing favipiravir to lopinavir/ritonavir combination by Cai et al. [27] and another to arbidol group by Chen et al. [28]. From the studies, it was found that favipiravir had better viral clearance compared to the lopinavir/ritonavir combination; and compared to the arbidol group, it had a higher recovery rate after 7 days of antiretroviral treatment. A phase III clinical trial of favipiravir in COVID-19 patients with non-severe symptoms is ongoing in Japan and the latest results showed a reduction in recovery time (11.9 days in the favipiravir group compared to 14.7 days in the placebo group) [29].

#### 2.3.3. Critical COVID-19 Patients

It is well known that the immunity of the human body decreases with age [30]. With the spread of the virus, elderly patients, especially those with comorbidities, are at a higher risk to suffer from COVID-19 symptoms [31]. Effective preventive measures are mandatory to reduce the risk of hospitalisation and death of the older population. One of such measures is the development of a safe and efficacious vaccine to protect the elderly from being infected by the virus [32]. Whilst good progress is being made in vaccine development, the standard operating procedure of COVID-19 management should be practised and retained as social responsibilities for the time being.

Most of the recommendations for the management of critically ill patients with COVID-19 are extrapolated from experiences with other causes of sepsis [33]. There is limited information to suggest that the critical care management of patients with COVID-19 should differ substantially from the management of other critically ill patients. However, taking special precautions to prevent environmental contamination by SARS-CoV-2 is warranted. As with any patient in the intensive care unit (ICU), successful clinical management of a patient with COVID-19 includes treating both the medical condition that initially resulted in ICU admission and other comorbidities and nosocomial complications.

## 3. Vaccine for SARS-CoV-2

### 3.1. Principles of Vaccine

The main structure of coronavirus includes a single-strand positive-strand nucleic acid (ssRNA), S protein, membrane protein (M), an envelope protein (E), and nucleocapsid protein (N). Amongst them, the S protein is responsible for recognising and binding to receptors on the surface of host cells and plays an important role in the first step of viral infection. Viral cell entry is facilitated by the fusion of viral and host cell membranes after the RBD of S protein binds to cellular ACE2. Studies have reported that the mechanical stability of SARS-CoV-2 RBD (250 pN) plays a vital role in increasing the spread of COVID-19 due to stronger intermolecular interactions as compared to RBD of SARS-COV (200 pN) [34]. The energetic studies of RBD have provided additional information regarding the stabilisation of S protein during the transition from close to open conformation before ACE2 recognition [35]. This transition prepares the virus for binding, fusion, and release of viral RNA into the cytoplasm and outperforms SARS-CoV in withstanding Brownian and cellular forces to maintain close contact with ACE2 for a post-fusion mechanism to occur. In addition, the M and E proteins are responsible for virus assembly, and the N protein plays an important role in RNA synthesis [36].

As the genome sequences of SARS-CoV-2 and SARS-CoV are highly similar, and they have the same cellular receptor ACE2, knowledge from the development of the SARS-CoV vaccine is important and served as the basis to outline the development of SARS-CoV-2 vaccines [37]. Studies have shown that vaccines targeting the RBD, S1, or S2 subunit of SARS-CoV-2 exhibit certain protective effects against COVID-19 [38]. Thus, the new coronavirus vaccines that are currently under research are generally designed and developed to destabilise the S protein and disrupt or weaken the RBD interactions.

On 24 January 2020, the Chinese Center for Disease Control and Prevention successfully isolated China’s first strain of new coronavirus [39]. Up to date, the global coronavirus vaccine research and development has covered various vaccine categories, including live virus and inactivated vaccines, subunit vaccines, vector vaccines, nucleic acid vaccines (mRNA vaccines and DNA vaccines), etc. [40].

#### 3.1.1. Live Virus Vaccines and Inactivated Vaccines

Live virus and inactivated vaccines with high immunogenicity that provide excellent stimulation to the immune system for antibody generation have been widely applied in the biomedical industry. Live virus vaccine is synthesised by the virus with reduced virulence or even non-virulence; whilst inactivated vaccines contain non-infectious intact virus with low- or non-pathogenicity, and thus, would not proliferate in vivo. Whilst live virus vaccines are developed with reduced virulence, they are closely monitored to ensure the reduced virulence is not restored [41] for the safe administration both during clinical trials and commercialised applications. Codagenix and the Serum Institute of India are developing a live serum attenuated vaccine based on the CodaVax technology under the Phase I trial [42].

#### 3.1.2. Subunit Vaccines

Subunit vaccines are composed primarily of non-genetic viral proteins or peptide fragments to trigger strong immune responses. The absence of an entire infectious virus in subunit vaccines increases the safety and eliminates the problem of virus inactivation or reversal of toxicity [41]. Studies have shown that most of the SARS-CoV-2 subunit vaccines target proteins, particularly the spike protein, or age proteins in specific areas; whilst some other subunit vaccines focus on N proteins. It aims to deliver antibodies and to contain human lymphocyte antigen (HLA) restricted T cell epitopes [43]. Israeli company MigVax is in the process of developing an oral anti-COVID-19 subunit vaccine based on previous research products [44]. At the same time, The Coalition for Epidemic Preparedness Innovations (CEPI) is working with the University of Queensland to develop a protein vaccine that uses “molecular clamps” to lock the coronavirus protein [45]. However, the study was terminated due to the potential risk of triggering Human Immunodeficiency Virus-false positives [46]. That being said, some other subunit vaccines have entered clinical trials, such as NVX CoV2373 in Phase III trial and SCB-2019 has completed Phase I trial [47].

#### 3.1.3. Vector Vaccines

The vector-based vaccine is a live attenuated vaccine that uses a modified safe virus such as adenovirus, measles, and influenza as a vector to express coronavirus proteins during the immunisation process. Vector vaccines are categorised into replicating and non-replicating vectors based on their replicating potential. Many of these viral vectors could not, or can only perform limited replication in human cells, and thus, have minimum safety concerns upon application. Viral vectors generally can perform quick synthesis of recombinants, verify protein expression, and accelerate the development of the immune system [41]. However, if the vaccine has been previously exposed to the targeted virus, the efficacy of the vaccine could be reduced due to the already existing immunity against the vector [48]. Currently, Hamilton, Massachusetts Institute of Technology (MIT), is cooperating with Oxford University to develop a chimpanzee adenovirus (serotype Y25) vectored SARS-CoV2 vaccine [49]. In addition to this, the Pasteur Institute, Themis, and the University of Pittsburgh Vaccine Research Center are developing a vaccine that expresses the SARS-CoV-2 S protein in a measles virus vector [50]. AZD1222, which expresses the native-like-spike protein of SARS-CoV-2, has been developed by the University of Oxford and AstraZeneca and is now in the combined Phase II/III trials [51]. 

#### 3.1.4. Nucleic Acid Vaccines (mRNA Vaccines and DNA Vaccines)

Nucleic acid vaccines can be produced in bulk and at a low cost without utilising live viruses. As these are not virus-containing vaccines, there is relatively low or no risk associated with virulence or infection upon application [52]. However, at the current stage of research, the distribution of DNA vaccines is rather challenging as the required dosage is relatively large. On the other hand, RNA vaccines face challenges with in vivo transfection efficiency [41]. Up to March 2021, the INO-4800 vaccine is conducting Phase II trials, and the primary developer has also planned to test the vaccine against the newly emerged variants [53]. The Moderna vaccine has been approved for use in Switzerland whilst Phase III trials are being conducted. Pfizer and BioNTech have developed four mRNA-based formulations, including two nucleoside modified mRNAs, where one contains uridine whilst the other contains self-amplifying RNA [54]. In addition, Tongji University in China, Imperial College London, Karolinska Institute, and Cobra Biologics are in collaborations to develop DNA vaccines named Comirnaty, also known as BNT162b2 [55], that is now in the combined Phase II/III trial [56]. Imperial College London has also explored a ‘self-amplifying’ RNA vaccine but the project was terminated on 27 January 2021 due to rapid virus mutation [57].

### 3.2. Current Vaccine Research Progress

#### 3.2.1. Time Frame

**Figure 1:**
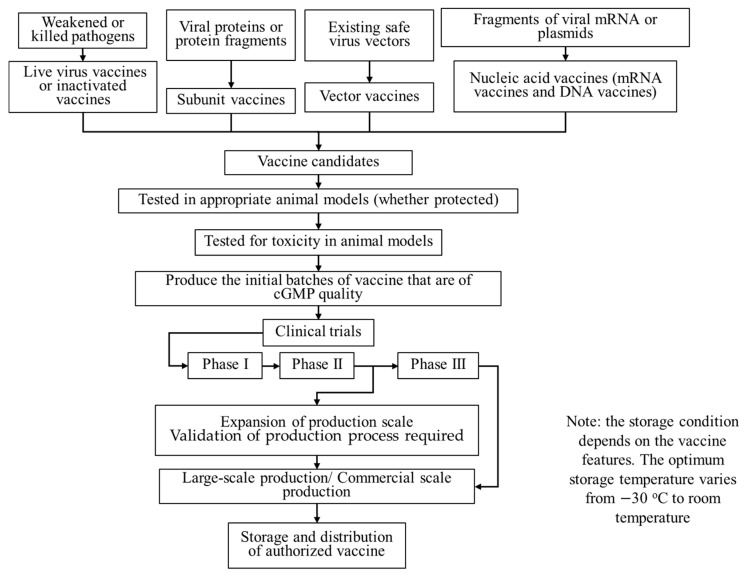
Flow chart of vaccine development from biological feedstock to clinical trials and logistic chain for vaccine distribution.

Under normal circumstances, the development of a new vaccine from pre-clinical trials until approval and licensing takes an average of 10 to 15 years. According to Amanat and Krammer (2020) [58], two significant steps are typically needed before a vaccine is tested in clinical trials. Vaccines are first tested for their ability to deliver protective immunity in suitable animal models, followed by the toxicity test of these vaccines on animals such as mice [59], ferrets [60], and monkeys [61] to ensure safety in application. Studies at the Chinese Academy of Sciences Wuhan Institute of Virology show that rhesus macaques infected with novel Coronavirus have symptoms similar to those of human COVID-19 patients [62]. Additionally, ferrets have lungs that are physiologically similar to those of humans, and thus, researchers can mimic certain aspects of COVID-19 in humans, including its spread, through ferret investigations [62]. Concerning the tests, Chinese researchers produced a purified inactivated SARS-CoV-2 vaccine candidate and tested it in mice, rats, and rhesus macaques to induce SARS-CoV-2-specific neutralising antibodies that proved successful [40]. After passing these two tests, the initial batches of this vaccine that are of current Good Manufacturing Practice (cGMP) quality are produced. Ideally, clinical trials will commence beginning with a Phase I trial to assess the safety of the candidate vaccine in humans, followed with a Phase II trial to determine the dosage to preliminarily prove the curative effect. The final Phase III trial is to demonstrate the efficacy and safety of the vaccine in a larger cohort. Eventually, the distribution of the vaccines adds 1 to 2 months to the whole timeline. The time frame for vaccine development to eventual marketing is illustrated in Figure 1.

Up to 20 March 2021, there are over 90 vaccine candidates in clinical development, with 22 of them undergoing Phase III clinical trials, 32 in Phase II trials, and 44 in Phase I trials [63,64,65]. WHO has set its success benchmark for COVID-19 vaccines with the highest at 70% efficacy under a protected duration of one year and the lowest threshold at 50% efficacy for 6 months [66]. The developed vaccines are bounded to be effective without raising safety concerns to the public that is receiving the vaccination. The vaccines will have to fulfil all regulatory requirements in terms of quality, efficacy, and safety before a marketing authorisation is granted. Table 1 summarises the ongoing Phase III trials of leading vaccines in March 2021, tabulated according to the timeline. Further details, including the official identifier number, trial period, trial population, study design, targeted outcome measure, and primary findings are tabulated in Table S1 under the supplementary document.

#### 3.2.2. Safety and Efficacy of Vaccines

By March 2021, thirteen vaccines (tabulated in Table 2) have been authorised for use in many countries. These vaccines have been demonstrated to be effective in preventing the infection of COVID-19 at varying efficacy. In terms of prevention of symptoms, the efficacy of the vaccine Comirnaty (BNT162b2) is 95%, followed by Moderna COVID-19 Vaccine (mRNA-1273) of 94.5% and inactivated vaccine BBIBP-CorV of 86%, whilst BNT162b2 (87%) is less effective in the prevention of severe disease, compared to the other two vaccines (100%) [92,93,94].

Clinical trial results of COVID-19 Vaccine AstraZeneca (AZD1222) [95] report the specific antibodies peaked by day 28 after vaccination and remained elevated until day 56. The mild or moderate adverse events of AZD1222 vaccination can be potentially reduced by paracetamol (acetaminophen) prophylaxis. The vaccination of CoronaVac is found to diminish with increased age; thus, an increased dosage is recommended for the elderly population [96]. Researchers report that the participants receiving the Sputnik V vaccine during clinical trials did not show unexpected adverse events after the first and second injections [97]. The 100% efficacy of the EpiVacCorona vaccine is evaluated based on its immunological and preventative efficacy from phase I and II trial results [98]. Both Sputnik V and EpiVacCorona vaccines exhibit positive results but further details of the clinical trials are yet to be made available. Convidicea, a novel coronavirus vaccine that incorporates adenovirus type 5 vector, was recently developed by China’s CanSino Biologics. Clinical trials showed that the specific T-cell response peaked at day 14 after vaccination whilst specific antibodies and neutralising antibodies increased significantly at day 14 and continued to peak for 28 days post-vaccination [99]. The trial reports 75–83% of vaccine recipients develop common mild or moderate adverse effects such as pain at the injection site and fever within 28 days post-vaccination, no serious adverse effects were recorded. Convidicea has been approved for use within the military in China and emergency use in Mexico. On 20 February 2021, Russia approved the use of a new vaccine named CoviVac; more than 3000 participants will be recruited for clinical trials and the studies are expected to be completed by November 2021 [100]. Following that, U.S. Food and Drug Administration issued emergency use authorisation for the Janssen COVID-10 Vaccine that allows distribution and use in individuals 18 years of age and older [101]. China National Medical Products Administration (NMPA) granted a conditional market authorisation to the new Wuhan-Sinopharm vaccine (the name is yet to be announced) in late February 2021 [102]. The vaccine was approved for emergency use for high-risk individuals in the country, particularly for healthcare workers. Phase I and II clinical trials reported that the Wuhan-Sinopharm vaccine demonstrated immunogenicity in two randomised placebo-controlled trials [103]. Results for the phase III trial of the Wuhan-Sinopharm vaccine, conducted in Peru, are yet to be made available. On the other hand, Uzbekistan authorised the ZF2001 vaccine for emergency use [104] on 1 March 2021. Research on this vaccine suggests that ZF2001 neutralises the South African variant of SARS-CoV-2 [105] and clinical trials are currently underway in China and Uzbekistan; and planned to begin in Malaysia, Ecuador, Indonesia, and Pakistan.

Apart from the efficacy, the accompanying adverse reactions also play an important role in evaluating the availability of these vaccines. Generally, there were no severe adverse reactions reported for people who have taken these vaccines and the mild side effects normally include headache, muscle/joint pain, fatigue, and so forth [103,106,107]. However, either efficacy or adverse effect needs to be evaluated solely for individuals with different ages, genders, and medical histories. For instance, Mahase suggested that people with a history of allergic reactions should avoid taking BNT162b2 vaccines, which may cause significant hypersensitivity.

**Table 2:** Authorised vaccines available for COVID-19 are arranged according to vaccine types in March 2021.

Vaccine Name	Vaccine Type	Primary Developers	Efficacy
Comirnaty, also known as BNT162b2	mRNA-based vaccine	Pfizer, BioNTech; Fosun Pharma	95% [108]
Moderna COVID-19 Vaccine, also known as mRNA-1273	mRNA-based vaccine	Moderna, U.S. Biomedical Advanced Research and Development Authority (BARDA), National Institute of Allergy and Infectious Diseases (NIAID)	94.5% [109]
CoronaVac	Inactivated vaccine	Sinovac	50–91% [110]
BBIBP-CorV	Inactivated vaccine	Beijing Institute of Biological Products; China National Pharmaceutical Group (Sinopharm)	79% [111]
Covaxin	Inactivated vaccine	Bharat Biotech, Indian Council of Medical Research (ICMR)	81% [112]
CoviVac	Inactivated vaccine	Chumakov Federal Scientific Center for Research and Development of Immune and Biological Products	Yet to be made available
Name yet to be announced	Inactivated vaccine	Wuhan Institute of Biological Products; China National Pharmaceutical Group (Sinopharm)	72.5% based on interim analysis [113]
AstraZeneca, also known as AZD1222 or Covishield in India	Vector vaccine (Adenovirus)	AstraZeneca, University of Oxford	70% [109]
Sputnik V, also known as Gam-Covid-Vac	Vector vaccine (Adenovirus Ad5 and Ad26)	Gamaleya Research Institute, Acellena Contract Drug Research and Development	92% [97]
Janssen COVID-19 Vaccine, also known as JNJ-78436735 or Ad26.COV2.S	Vector vaccine (Adenovirus Ad26)	Janssen Biotech Inc.—Janssen Pharmaceutical Company of Johnson & Johnson	76.7–85.4% for molecularly confirmed severe/critical COVID-19 patients [114].
Convidicea, also known as Ad5-nCoV	Vector vaccine (Adenovirus Ad5)	CanSino Biologics	65.7% in prevention and 90.98% in terminating severe symptoms (interim analysis) [115]
ZF2001	Vector vaccine (Recombinant vaccine)	Anhui Zhifei Longcom Biopharmaceutical, Institute of Microbiology of the Chinese Academy of Sciences	Yet to be made available
EpiVacCorona	Subunit vaccine	Federal Budgetary Research Institution State Research Center of Virology and Biotechnology	100% (Based on phase I and II trials) [98]

### 3.3. Challenges Encountered in Developing Vaccines and Current Progress

#### 3.3.1. Efficacy and Safety

More than 100 vaccines are being developed in various countries [41], and the establishment of efficacious and safe vaccines is urgently needed to curb the current pandemic. As clinical trials and evaluation of vaccine efficacy by the vaccine dossiers are expedited, post-marketing surveillance will play an important role in discovering any new adverse reaction to the vaccine in post-authorisation safety studies (PASS) and real-world evidence reporting. Currently, the most common adverse side reactions to the BNT162b2 mRNA-based vaccine are pain at the injection site, fatigue and headaches though there have been several reports of subjects having an anaphylactic reaction after receiving the vaccine [56,116]. Hence, the Medicines and Healthcare products Regulatory Agency (MHRA) guidelines state that any person with a history of immediate onset anaphylaxis to a vaccine, medicine, or food should not receive the BNT162b2 mRNA-based vaccine [117].

#### 3.3.2. Emergence of Coronavirus Variants

Variants of coronavirus have been detected in recent months due to virus mutation and adaption to increase its survival [118]. B.1.1.7 (or VUI 202012/01) is one of the many variants that exhibit a much higher transmission rate than the original virus and that of the other variants at 71% (95% CI: 0.67 to 0.75) [119]. B.1.1.7, which is the dominant variants in the United Kingdom, is suspected to be associated with a higher risk of death as compared to other variants due to spontaneous and frequent conformational change in the spike protein with increased transmissibility [120]. Similarly, P.1 variant emerged with three mutations in the S protein RBD at K417T, E484K, and N501Y [121]. These mutations are found to affect the antigenic profile and transmissibility by disrupting the ability of antibody generation from previous natural infection or through vaccination. In South Africa, variant B.1.351 emerged independently of B.1.1.7 and has similar mutations in the spike protein. However, there is no current evidence to suggest that this variant has any impact on neutralising antibodies or affecting the disease severity [122].

A vaccine escape must not happen in the coming months in which the infection rate would increase due to ineffective vaccines against mutated viruses. If this were to occur, the vaccines will have to be updated or redesigned regularly and yearly vaccine immunisation against the coronavirus is expected.

#### 3.3.3. Vaccine Distribution Challenges

The frontrunners in late-stage vaccine development, with reported interim analysis results of the vaccine being 95% effective in preventing COVID-19, is from the phase III clinical trials of the BNT162b2 mRNA-based vaccine candidate from Pfizer and BioNTech. The vaccine requires storage at −70 °C throughout the distribution process from the manufacturer’s store to its intended destination [123]. Whilst stability studies at a temperature of 2 to 8 °C (36 to 46°F) allow the vaccine to remain stable for 5 days, it requires critical and meticulous planning when the vaccine is being transferred from the specialised freezers to the healthcare professional fridge to prevent the vaccine from losing its effectiveness due to suboptimal storage condition. These storage conditions pose a challenge to vaccine distribution particularly to many parts of the world that are unable to store these vaccines at such low temperatures, as only specialised freezers can produce such ultra-cold storage conditions. This means that cities or areas that do not have the capability of an ultra-cold storage system will have to wait longer for other vaccine candidates that are stored at milder conditions.

The Moderna vaccine candidate reported their preliminary trial results of a 94% effectiveness of the vaccine in preventing COVID-19 [124]. The vaccine is expected to be stored at a less stringent storage condition, as compared to Pfizer and BioNTech mRNA-based vaccines, at −20 °C for 6 months and 2 to 8 °C for 30 days, which poses less of a challenge as most hospitals and pharmacies have facilities to store the vaccine. An ideal vaccine distribution pathway is already established in all the countries especially towards the challenging rural areas.

## 4. Conclusions

Whilst the development of vaccines for COVID-19 is a long and tedious, yet urgent process, promising progress has been made in recent months. Multiple vaccines have been prepared and extensive preclinical research has been conducted. It begins with animal model verification and those successful proceeding to clinical trials in humans. Under normal circumstances, these vaccines can be approved for marketing only after the phase III clinical trials have fully confirmed that the vaccines are safe and effective. However, due to the urgency of this pandemic, COVID-19 vaccines have been given conditional, emergency, or temporary use authorisation with high surveillance on their efficacy and safety profile post-authorisation. Whilst such progress is promising, it is necessary to strengthen the popularisation of prevention knowledge and containment measures to collectively control the spread of the pandemic with measures such as social distancing, frequent hand washing, and mask-wearing in public areas. The world population will have to adapt to these new preventive measures in order to flatten the pandemic curve, at least until safe and effective vaccines are made available to the general public, particularly those most vulnerable.

## Figures and Tables

**Table 1:** Timeline of phase III clinical trials of leading vaccines up to March 2021. Specific codes, i.e., 1a, 1b, etc., are assigned to each registered clinical trial where the details and findings are tabulated in Table S1 under the supplementary document.

No.	Primary Developers	Year and Month	2020									2021		
		Vaccine Name	04	05	06	07	08	09	10	11	12	01	02	03
1	Pfizer, BioNTech; Fosun Pharma	Comirnaty [67,68,69]	1a			1b							1c	
2	Moderna, U.S. Biomedical Advanced Research and Development Authority (BARDA), National Institute of Allergy and Infectious Diseases (NIAID)	mRNA-1273 [70,71]				2a					2b			
3	Sinovac	CoronaVac [72,73,74]				3a	3b	3c						
4	Beijing Institute of Biological Products; China National Pharmaceutical Group (Sinopharm)	BBIBP-CorV [75]				4a								
5	Bharat Biotech, Indian Council of Medical Research (ICMR)	Covaxin [76]							5a					
6	Chumakov Federal Scientific Center for Research and Development of Immune and Biological Products	CoviVac												6a
7	Wuhan Institute of Biological Products; China National Pharmaceutical Group (Sinopharm)	Name yet to be announced				7a								
8	AstraZeneca, University of Oxford	AZD1222 [77]		8a										
9	Gamaleya Research Institute, Acellena Contract Drug Research and Development	Sputnik V [78]						9a						
10	Janssen Biotech Inc. - Janssen Pharmaceutical Company of Johnson & Johnson	Janssen COVID-19 Vaccine [79,80]						10a		10b				
11	CanSino Biologics	Convidicea [81]					11a							
12	Anhui Zhifei Longcom Biopharmaceutical, Institute of Microbiology of the Chinese Academy of Sciences	ZF2001 [82]									12a			
13	Federal Budgetary Research Institution State Research Center of Virology and Biotechnology	EpiVacCorona [83]								13a				
14	Medicago	CoVLP [84]								14a				
15	CureVac, GlaxoSmithKline (GSK)	CVnCoV [85]									15a			
16	AnGes, Inc., Osaka University and Takara Bio.	AG0302-COVID19 [86]									16a			
17	Clover Biopharmaceuticals	SCB-2019 [87]												17a
18	Institute of Medical Biology at the Chinese Academy of Medical Sciences	Name yet to be announced [88]										18a		
19	Zydus Cadila	ZyCoV-D [89]										19a		
20	ReiThera, Lazzaro Spallanzani National Institute for Infectious Diseases	GRAd-COV2 [90]												20a
21	Finlay Vaccine Institute	Soberana 2												21a
22	Research Institute for Biological Safety Problems	QazCovid [91]												22a

## Data Availability

Not applicable.

## Supplementary Materials

The following are available online at https://www.mdpi.com/article/10.3390/vaccines9040349/s1.
