# Supplementary material for: The COVID-19 Vaccines: Recent Development, Challenges and Prospects
Source: Vaccines (Basel). 2021 Apr 5;9(4):349. doi: 10.3390/vaccines9040349 (PMC8067284; doi:10.3390/vaccines9040349)
Supplement: Supplementary file 1 [file vaccines-09-00349-s001.pdf]

Table S1: Details of phase III clinical trials of different vaccines up to March 2021.

Clinical trial code (Identifier No.)	Trial period	Population	Study design	Targeted outcome measures /Primary findings	Remarks
1a (NCT04368728) [1]	April 2020 – January 2023	43998	Randomised, placebo-controlled, observer-blind, dose-finding	Investigate local reactions, systemic events, adverse events (AEs), serious AEs (SAEs) and hematology [Time frame of 7 days after dose 1 and dose 2, and through 6 months after the last dose]<br>Measuring antibody level by Neutralising antibody Geometric mean titers (GMT), Geometric Mean Fold Rise (GMFR), Geometric Mean Ratio (GMR)	95% effective in preventing COVID-19 in participants without evidence of prior SARS-CoV-2 infection based on interim analysis
1b (NCT04713553) [2]	February 2021 – April 2022	1530	Randomised, observer-blind	Investigate antibody level, local reactions, systemic events, AEs and SAEs [Time frame of 7 days after dose 1 and dose 2, and through 1 month after the last dose]<br>Measuring antibody level by using Geometric Mean Ratio (GMR), GMFR and Geometric Mean Concentrations (GMCs)	
1c (NCT04754594) [3]	February 2021 – June 2022	4000	Randomised, placebo-controlled, observer-blind, 4000 healthy pregnant women 18 years of age or older	Investigate local reactions, systemic events, inferiority of immune response, AEs and SAEs [Time frame of 7 days after dose 1 and dose 2, and through 6 months after the last dose]	
2a (NCT04470427) [4]	July 2020	30420	Randomised,	Investigate AEs, medically attended AEs	Preliminary trial

	– October 2022		stratified, observer-blind, placebo-controlled, adults aged 18 years and older	(MAAEs), solicited local and systemic adverse reactions (ARs), unsolicited AEs and SAEs. [Time frame of day 8 to day 36, day 28, day 43 to day 759]<br>Measuring antibody level by using GMT and GMFR	result shows the vaccine is 94% effective in preventing in COVID-19 as announced on November 16 [5].
2b (NCT04649151) [6]	December 2020 – June 2022	3000	Randomised, observer-blind, placebo-controlled, adolescents 12 to <18 years of age	Investigate ARs, AEs, SAEs, MAAEs, AESI and number of participants infected. [Time frame of day 1, day 8, day 36, day 57, day 209, day 394]<br>Measuring antibody level by geometric mean value. [Time frame of day 1, day 57, day 209, day 394]	
3a (NCT04456595) [7]	July 2020 – February 2022	12688	Double-blind, randomised, placebo-controlled	Investigate the frequency of AEs and incidence of COVID-19 cases. [Time frame of two weeks after the first dose up to one year after first dose, 7 days up to 28 days after each immunisation and from first vaccination up to one year after first dose]	
3b (NCT04508075) [8]	August 2020 - September 2021	1620	Randomised, observer-blind, placebo-controlled, 18-59 years of age	Investigate incidence of COVID-19 cases, SAEs, Local reaction and systemic events. [Time frame of 30 minutes to 14 days after each vaccination, 14 days to 6 months after the second dose]<br>Measuring the antibody level by using seroconversion (SC) rate and seropositive rate. [Time frame of 14 days up to 6 months after two doses of vaccination]	

3c (NCT04582344) [9]	September 2020 – April 2021	13000	Randomised, double-blind, placebo-controlled	Investigate the protection indexes and safety indexes. [Time frame of 2 weeks after the second dose of vaccination, 7 days after each dose, 28 days up to 1 year after second dose]<br>Measuring the antibody level by using SC rate and seropositive rate. [Time frame of 14 days up to 28 days after two doses of vaccination]	
4a (ChiCTR2000034780) [10]	July 2020 – July 2021	15000	Randomised, parallel assignment of vaccine for adults 18 years and above	To evaluate the protective effect 14 days after 2 doses of immunisation of preventing severe cases of SARS-CoV-2 pneumonia and deaths accompanied by COVID-19.	Four-fold growth rate and antibody level (GMT, GMI) of serum antibody against COVID-19
5a (CTRI/2020/11/028976) [11]	November 2020 – November 2021	25800	Randomised, parallel assignment of vaccine for adults 18 years and above	To evaluate the efficacy of BBV152B to prevent symptomatic COVID-19 (Virologically confirmed-(RT-PCR positive) which include any participant who meets the Case Definitions for Symptomatic Endpoint and Severe Symptomatic COVID-19 [Time frame of 42 days to 12 months]	
6a	March 2020		Further details yet to be made available		
7a	July 2020		Further details yet to be made available		

8a (NCT04400838) [12]	May 2020 – September 2021	12390	Randomised, parallel assignment of vaccine for adults 18 years and above	Assess the efficacy and safety of the candidate ChAdOx1 nCoV-19 against COVID-19 in adults. [Time Frame: Study duration of 12 months from last vaccination]<br>Measure the number of virologically confirmed (PCR or NAAT positive) symptomatic cases of COVID-19 and occurrence of serious adverse events (SAEs) throughout the study duration	
9a (NCT04530396) [13]	September 2020 – May 2021	33758	Randomised, parallel assignment of vaccine for adults 18 years and above	Investigate the percentage of trial subjects with coronavirus disease 2019 (COVID-19) developed within 6 months after the first dose [Time Frame: through the whole study, an average of 180 days] and the severity of the clinical course of COVID-19 [Time Frame: through the whole study, an average of 180 days]	Interim analysis reported that the vaccine is 92% effective based on 20 identified COVID cases.
10a (NCT04436276) [14]	July 2020 – February 2024	60000	Randomised, parallel assignment of vaccine for adults 18 years and above		The company pauses its Phase 3 trial to investigate an adverse reaction in a volunteer.
10b (NCT04614948) [15]	November 2020 – May 2023	30000	Randomised, parallel assignment of vaccine for adults 18	Investigate the safety and efficacy of a two-dose vaccine	

			years and above		
11a (NCT04526990) [16]	September 2020 – January 2022	40000	Randomised, parallel assignment of vaccine for adults 18 years and above	Investigate the incidence of virologically confirmed COVID-19 disease, and occurrence of solicited and unsolicited AEs [Time frame of 28 days to 12 months post-vaccination and day 0 to 12 months after vaccination, respectively]<br>Investigate the cell-mediated immune profile, the immunogenicity of S-RBD IgG and SC antibody [Time frame of day 28 post vaccination]	
12a (NCT04646590) [17]	December 2020 – April 2022	29000	Randomised, parallel assignment of vaccine for adults 18 years and above with 25% subjects aged 60 and above	Investigate immune persistence by sampling IgG and RBD protein-binding antibody [Timeframe of 14 days and 6 months after full course of vaccination]	
13a (NCT04780035) [18]	November 2020 – September 2021	3000	Randomised, parallel assignment of vaccine for adults 18 years and above	Investigate tolerability and AEs upon dual dose of vaccination [Timeframe of day 0 to 9 months of study]<br>Investigate humoral and cell-mediated immune response by evaluating specific neutralising and GMT antibodies.	Prophylactic efficacy of the vaccine under study is ≥50% compared to a placebo
14a (NCT04636697) [19]	November 2020 –	30918	Randomised, parallel	Investigate immediate, solicited, unsolicited and serious AEs [Time frame of	

	April 2202		assignment of vaccine for adults 18 years and above	30 minutes, 7 days, 21 days and 386 days, respectively]. Measuring antibody response by using GMT, SC, GWFR and IgG at day 21, 42, 201, 386.
15a (NCT04652102) [20]	December 2020 – March 2023	36500	Randomised, parallel assignment of vaccine for adults 18 years and above	Investigate number of participants and intensity grading of AEs per FDA toxicity grading [Time frame: 29 to 393 days] Recording number of participants with serum vital neutralising antibodies and experience seroconversion to SARS-CoV-2 virus [Time frame of days 1, 29, 43, 57, 120, 211 and 393]
16a (NCT04655625) [21]	November 2020 – March 2022	500	Randomised, parallel assignment of dual vaccine dosage in healthy adults	Investigate frequency and severity of treatment-emergent AE [Time frame of first vaccination to 4th week after second vaccination] Investigate change in GMT and IgG subclasses (IgG1 and IgG2) of antibodies; change in neutralising activity and IFN-gamma production against pseudovirus of SARS-CoV-2.
17a (NCT04672395) [22]	March 2021 – July 2022	22000	Randomised, parallel assignment of dual vaccine dosage in adults 18 years and above	Investigate numbers of participants with first occurrence of COVID-19 after vaccination [Time frame of 14 days to 1 year after second dose] Investigate participants with solicited, unsolicited and serious AEs [Time frame of day 29, 43 and 389 days, respectively] Investigate antibody response with GMT, GMFR and SC [Time frame of day 1, 22, 35,

				205 and 389]
18a (NCT04659239) [23]	January 2021 – July 2022	34020	Randomised, parallel assignment of vaccine for adults 18 years and above	Investigate the incidence of COVID-19 cases after both doses of vaccination and solicited AEs [Time frame of 14 days to 1 year after second dose]<br>Investigate positive rates of GMT and IgG neutralising antibodies as well as specific T cell assay and occurrence of Antibody-Dependent Enhancement (ADE) [Timeframe of 6 months and 12 months after whole course immunisation]
19a (CTRI/2020/07/026352) [24]	January 2021 – no date specified	1048	Randomised, adaptive, 18-55 years of age	Investigate the safety of vaccine.<br>Measure antibody response.
20a (NCT04791423) [25]	March 2021 – April 2022	10300	Randomised, stratified, observer-blind, placebo-controlled, aged 18 years and older	Investigate AEs, SAEs, MAAEs, AESI, local and systemic solicited AEs and incidence of COVID-19 cases. [Time frame of day 29 to day 730, 7 days after each dose]<br>Measuring antibody response by using GMTs and GMFRs. [Time frame of day 1 to day 730]
21a	March 2021		Further details yet to be made available	
22a (NCT04691908) [26]	December 2020 – July 2021	3000	Multicenter, randomised, blind, placebo-controlled	Investigate AEs, SAEs and seroconversion. [Time frame of day 0, 21, 42, 90, 180, 1-2 weeks after each dose, throughout the study]<br>Measuring the antibody response and

	frequency of confirmed cases. [Time frame of day 0, 21, 42, 90, 180, 1-2 weeks after each dose, throughout the study,]	

References

1. U.S. National Library of Medicine, *Study to Describe the Safety, Tolerability, Immunogenicity, and Efficacy of RNA Vaccine Candidates Against COVID-19 in Healthy Individuals*. 2020.
2. U.S. National Library of Medicine, *A Phase 3 Study to Evaluate the Safety, Tolerability, and Immunogenicity of Multiple Production Lots and Dose Levels of BNT162b2 Against COVID-19 in Healthy Participants*. 2021.
3. U.S. National Library of Medicine, *Study to Evaluate the Safety, Tolerability, and Immunogenicity of SARS CoV-2 RNA Vaccine Candidate (BNT162b2) Against COVID-19 in Healthy Pregnant Women 18 Years of Age and Older*. 2021.
4. U.S. National Library of Medicine, *A Study to Evaluate Efficacy, Safety, and Immunogenicity of mRNA-1273 Vaccine in Adults Aged 18 Years and Older to Prevent COVID-19*. 2020.
5. Diamond, M.S. and Pierson, T.C., The challenges of vaccine development against a new virus during a pandemic. *Cell Host & Microbe,* **2020**. 27(5): p. 699-703.
6. U.S. National Library of Medicine, *A Study to Evaluate the Safety, Reactogenicity, and Effectiveness of mRNA-1273 Vaccine in Adolescents 12 to <18 Years Old to Prevent COVID-19 (TeenCove)*. 2020.
7. U.S. National Library of Medicine, *Clinical Trial of Efficacy and Safety of Sinovac's Adsorbed COVID-19 (Inactivated) Vaccine in Healthcare Professionals (PROFISCOV)*. 2020.
8. U.S. National Library of Medicine, *Efficacy, Safety and Immunogenicity Study of SARS-CoV-2 Inactivated Vaccine (COVID-19)*. 2020.
9. U.S. National Library of Medicine, *Clinical Trial For SARS-CoV-2 Vaccine (COVID-19)*. 2020.
10. Chinese Clinical Trial Registry, *A Phase III clinical trial for inactivated novel coronavirus pneumonia (COVID-19) vaccine (Vero cells)*. 2020.
11. India National Institute of Medical Statistics, *A Phase 3, Randomized, Double-blind, Placebo-controlled, Multicenter Study to Evaluate the Efficacy, Safety, Immunogenicity, and Lot-to-Lot consistency of BBV152, a Whole virion Inactivated Vaccine in Adults greater than or equal to 18 Years of Age.* 2020.
12. U.S. National Library of Medicine, *Investigating a Vaccine Against COVID-19*. 2020.

13. U.S. National Library of Medicine, *Clinical Trial of Efficacy, Safety, and Immunogenicity of Gam-COVID-Vac Vaccine Against COVID-19 (RESIST)*. 2020.
14. U.S. National Library of Medicine, *A Study of Ad26.COV2.S in Adults (COVID-19)*. 2020.
15. U.S. National Library of Medicine, *A Study of Ad26.COV2.S for the Prevention of SARS-CoV-2-mediated COVID-19 in Adults (ENSEMBLE 2)*. 2020.
16. U.S. National Library of Medicine, *Phase III Trial of A COVID-19 Vaccine of Adenovirus Vector in Adults 18 Years Old and Above*. 2020.
17. U.S. National Library of Medicine, *A Phase III Clinical Trial to Determine the Safety and Efficacy of ZF2001 for Prevention of COVID-19*. 2020.
18. U.S. National Library of Medicine, *Study of the Tolerability, Safety, Immunogenicity and Preventive Efficacy of the EpiVacCorona Vaccine for the Prevention of COVID-19*. 2020.
19. U.S. National Library of Medicine, *Study of a Recombinant Coronavirus-Like Particle COVID-19 Vaccine in Adults*. 2020.
20. U.S. National Library of Medicine, *A Study to Determine the Safety and Efficacy of SARS-CoV-2 mRNA Vaccine CVnCoV in Adults for COVID-19*. 2020.
21. U.S. National Library of Medicine, *Phase II / III Study of COVID-19 DNA Vaccine (AG0302-COVID19)*. 2020.
22. U.S. National Library of Medicine, *A Controlled Phase 2/3 Study of Adjuvanted Recombinant SARS-CoV-2 Trimeric S-protein Vaccine (SCB-2019) for the Prevention of COVID-19 (SCB-2019)*. 2021.
23. U.S. National Library of Medicine, *The Efficacy, Safety and Immunogenicity Study of Inactivated SARS-CoV-2 Vaccine for Preventing Against COVID-19*. 2020.
24. India National Institute of Medical Statistics, *A prospective, randomized, adaptive, phase I/II clinical study to evaluate the safety and immunogenicity of Novel Corona Virus -2019-nCov vaccine candidate of M/s Cadila Healthcare Limited by intradermal route in healthy subjects*. 2020.
25. U.S. National Library of Medicine, *Study of GRAd-COV2 for the Prevention of COVID-19 in Adults (COVITAR)*. 2021.
26. U.S. National Library of Medicine, *Immunogenicity, Efficacy and Safety of QazCovid-in® COVID-19 Vaccine*. 2020.
